# The Sanitarian

**Published:** 1876-01

**Authors:** 


*The Sanitarian.*—A monthly Journal, Edited by A. N. Bell, M. D. Publication office 235 Broadway, N. Y. Terms, $3.00 per year, or 30 cents a number.

This useful *Journal* should be read by all interested in the laws of health. It is admirably conducted and presents monthly a quantity of reading matter which cannot fail to interest and instruct the intelligent and thoughtful observer. Every head of a family is interested in the subjects so clearly and forcibly brought to the public notice in this useful periodical.

